# Translation and validation of the German version of the Chronic Otitis Media Questionnaire-12 (COMQ-12): a psychometric analysis

**DOI:** 10.1186/s40359-026-04713-0

**Published:** 2026-05-16

**Authors:** Michael Knoke, Marcus Neudert, Thomas Zahnert, Susen Lailach

**Affiliations:** 1 Charité Universitätsmedizin Campus Virchow-Klinikum, Campus Charité Mitte, Augustenburger Platz 1, Berlin, 13353 Germany; 2 Universitätsklinikum Carl Gustav Carus Dresden, Dresden, Germany

**Keywords:** Quality of life, Questionnaire, Chronic otitis media, Cholesteatoma, Patient-reported outcomes, Response shift

## Abstract

**Background:**

The Chronic Otitis Media Questionnaire-12 (COMQ-12) is a disease-specific health-related quality of life (HRQoL) instrument originally developed in English for patients with chronic otitis media (COM). This study aimed to translate the COMQ-12 into German and to validate the resulting German version, and assess its psychometric properties.

**Methods:**

The COMQ-12 underwent forward–backward translation into German. Seventy-nine patients with COM who underwent middle ear surgery were prospectively enrolled. HRQoL was assessed preoperatively and 6 months postoperatively via the German COMQ-12. Additionally, pure tone audiometry was performed at both time points. Psychometric properties, including internal consistency, test–retest reliability, discriminant validity, agreement validity, responsiveness, and response shift, were evaluated.

**Results:**

The German COMQ-12 demonstrated high internal consistency both preoperatively (Cronbach’s α = 0.87) and postoperatively (Cronbach’s α = 0.84). The test–retest reliability was high (r = 0.89). It significantly discriminated between COM patients and healthy controls (*p < *0.001) and showed strong convergent validity with a global health-specific question (*r* = 0.64, *p < *0.001). The questionnaire exhibited high responsiveness (standardized response mea*n =* -0.80) with minimal response shift effects (effect size = 0.30). The correlations with the audiometric parameters were weak (*r* = 0.11–0.16).

**Conclusions:**

The German version of the COMQ-12 has satisfactory psychometric properties and can be recommended for assessing HRQoL in German-speaking population with COM. Its suitability for evaluating symptom-related quality of life is due to its symptom-focused design and high level of responsiveness. Its low response shift also makes it ideal for longitudinal monitoring of treatment outcomes.

## Background

Chronic otitis media (COM) is a long-standing inflammatory condition of the middle ear associated with symptoms such as recurrent otorrhea, hearing loss, tinnitus, and vertigo [1]. These symptoms often result in significant impairments in daily functioning, social participation, and overall health-related quality of life (HRQoL). While pure-tone audiometry is essential for assessing hearing thresholds, it fails to capture the broader physical, emotional, and social consequences of the disease. As a result, patient-reported outcome measures (PROMs) have become indispensable tools in otology for evaluating treatment outcomes and understanding the patient perspective.

Among disease-specific PROMs for COM, the Chronic Otitis Media Questionnaire 12 (COMQ-12) is one of the most widely used instruments internationally [2–5]. The COMQ-12 [6] consists of 12 items that assess symptom severity, functional limitations, and psychosocial impact. It yields a single total score, making it practical and time-efficient for both clinical use and research. While the absence of subscales limits detailed domain-specific analysis, it simplifies interpretation, reduces statistical complexity, and enhances applicability in international studies and routine care.

The COMQ-12 was developed through item reduction and integration of elements from several earlier PROMs [6], most notably the Chronic Otitis Media Outcome Test-15 (COMOT-15), the Chronic Otitis Media-5 (COM-5), and the Chronic Ear Survey (CES). The CES is used to evaluate symptom severity, activity restrictions, and healthcare use [7]. Although more detailed and lengthier, it served as an important conceptual foundation for the COMQ-12. A validated German version of the CES [8] is available, allowing for direct psychometric comparisons across instruments. The COMOT-15 was the first disease-specific HRQoL instrument developed in German language [9]. It includes 15 items, providing a total score and three subscales: “Ear Symptoms,” “Hearing Function,” and “Mental Health.” Although it allows for a more differentiated evaluation of disease burden, its multidimensional format increases analytical effort and limits comparability with instruments such as the COMQ-12, which follow a more concise structure. The COM-5 is a validated questionnaire designed to assess health-related quality of life in children with chronic otitis media. It captures the physical, emotional, and social impacts of a condition from the perspectives of both the child and their caregivers [10].

Despite its widespread international use and availability in multiple languages—including French, Spanish, Italian and Portuguese [3, 11–13]—a validated German version of the COMQ-12 has been lacking until now. This has limited its adoption in German-speaking populations and hindered participation in multinational clinical studies using this instrument as a standard.

The present study aims to close this gap by validating the German translation of the COMQ-12 and comparing its psychometric performance with that of the COMOT-15 and the CES. The analysis includes internal consistency, test–retest reliability, construct and discriminant validity, responsiveness, and correlations with audiometric parameters. The results will help guide the selection and use of PROMs in both national and international contexts in the assessment of chronic otitis media, and enables standardized international comparisons in German-speaking clinical and research settings.

## Methods

The study was approved by the Ethics Committee of the University Hospital Dresden (EK 268072014). A total of 79 patients (mean age 48.6 ± 14.9 years; 50 female [63%], 29 male [37%]) who underwent COM surgery (mucosal: *n =* 41 [52%]; cholesteatoma: *n =* 38 [48%]) at Dresden University Hospital between May 2016 and May 2018 were prospectively recruited. Comorbidities (e.g., diabetes mellitus, chronic rhinosinusitis) were documented prospectively but not used as exclusion criteria to reflect real-world tertiary care cohort. Patients under 18 years of age, noncompetent patients, and patients who had to undergo another ear surgery during the study period were excluded.

All patients underwent pure tone audiometry prior to surgery and again 6 months postoperatively for follow-up. Audiological evaluation was based on the four-frequency pure tone average (4FPTA) for air conduction thresholds and the air–bone gap (ABG), which was calculated across the frequencies 0.5, 1, 2, and 4 kHz and included bilateral assessment of both ears. For correlation analyses with COMQ-12 scores, the 4FPTA of the affected ear was used, as the questionnaire specifically asks about symptoms and hearing difficulties in the diseased ear. For the description of overall hearing status and functional hearing disability at the group level, bilateral audiometric data including the better-hearing ear were reported. At both time points, HRQoL was assessed via the German version of the COMQ-12, the German CES and the COMOT-15. An additional assessment of quality of life with the COMQ-12 was conducted one week after the follow-up examination.

As a control group, otologically healthy subjects with self-reported normal hearing were included after being informed about the study and its procedures.

### Chronic Otitis Media Questionnaire-12 (COMQ-12)

The COMQ-12 was developed in 2014 from the COMOT-15, CES, and COM-5 in English after a long development and evaluation phase and integrates physical aspects of the three measurement instruments in patients with chronic otitis media [6].

The content validity of the German COMQ-12 was established through a structured, multistep process adhering to COSMIN (Consensus-based Standards for the selection of health Measurement Instruments) guidelines [14]. While the original English instrument was developed based on extensive literature review and patient feedback to ensure coverage of relevant symptom domains [6], the validity of the German version was independently verified.

First, the questionnaire underwent a rigorous forward–backward translation procedure. Two native German speakers independently translated the instrument, followed by a blinded back-translation by two native English speakers to ensure conceptual equivalence.

Second, three experienced otologic surgeons independently evaluated each translated item for relevance and appropriateness, confirming that the content accurately reflected the clinical constructs of the original instrument. Third, to assess comprehensibility and comprehensiveness from patient’s perspective, a cognitive debriefing was conducted with a pilot group of five patients with chronic otitis media. These patients evaluated each item for relevance (4-point scale), comprehensibility, and comprehensiveness of coverage. All items achieved high relevance ratings (≥ 3.5/4) and were easy to understand. Patients confirmed that no important disease-related aspects were missing. This combination of expert appraisal and patient testing supports the content validity of the German version.

**Fig. 1:**
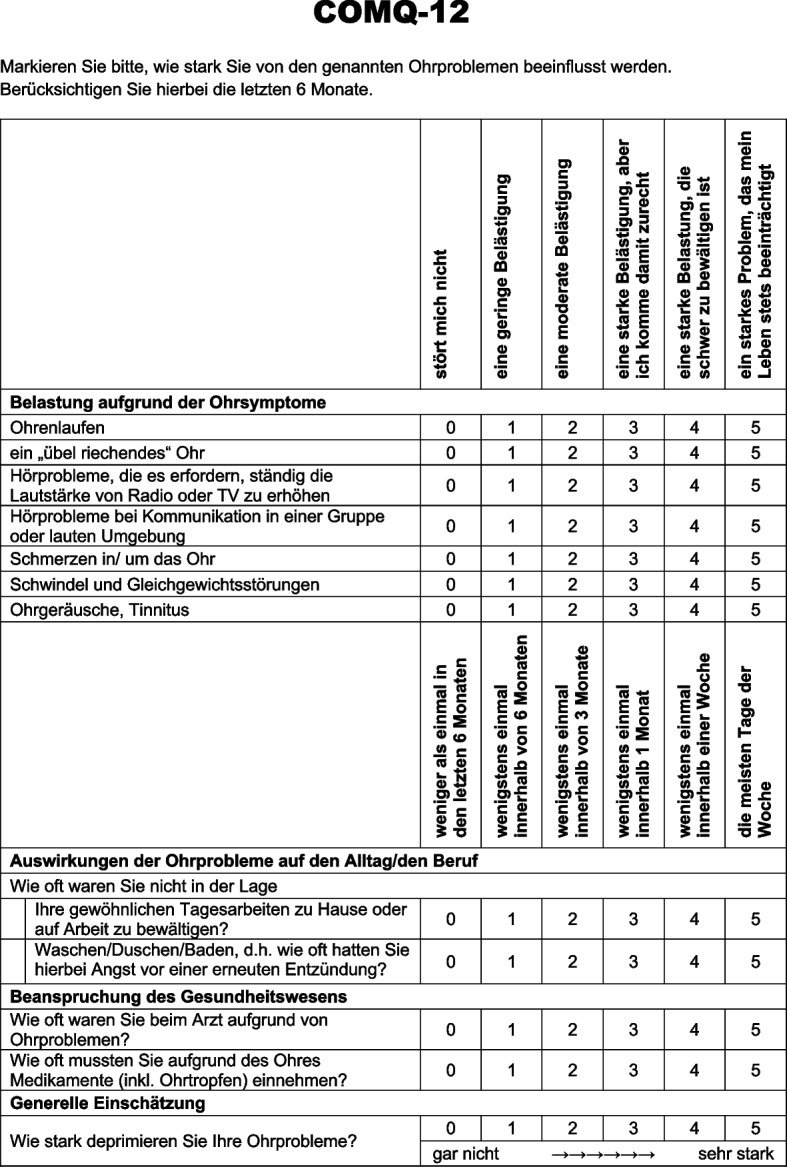
COMQ-12 in the German language

To validate the German COMQ-12 (Fig. 1), comprehensive analyses of the tool’s reliability, validity, and responsiveness were conducted.

The test consists of twelve items and offers Likert-scaled six-point response options (0—5) [6]. The total score is determined via summation. A higher value is associated with poorer quality of life [6]. Subscores are not provided.

### Chronic Otitis Media Outcome Test 15 (COMOT-15)

The COMOT-15 is a disease-specific questionnaire developed and validated to assess HRQoL in patients with COM in the German language [9]. It consists of 15 items, each rated on a 5-point Likert scale. In addition to an overall score (calculated from items 1—13), the questionnaire generates three subscores: “Ear Symptoms” (items 1—6), “Hearing Function” (items 7—9), and “Mental Health” (items 10—13). Furthermore, item 14 provides a general assessment of disease-specific HRQoL, whereas item 15 captures the frequency of medical resource utilization. The COMOT-15 scores are transformed to a scale ranging from 0 to 100, with 0 indicating the least impairment of HRQoL [9].

### Chronic Ear Survey (CES)

The CES is a psychometric assessment tool consisting of 13 items designed to evaluate the frequency, duration, and severity of symptoms associated with chronic otitis media, thereby providing a measure of HRQoL. The responses to these items vary, utilizing either frequency ratings of four- to six-point Likert scales. The instrument is organized into three subscores: “Limitations in activities” (covering questions a1 to a3), “Symptoms” (questions s1 to s7), and “Use of medical resources” (questions m1 to m3). Each response is converted to a scale ranging from 0 to 100. To calculate the total CES score, the values for each subscore are first averaged by dividing the sum of their respective item scores by the number of items in that subscore. These three averaged subscores are then added together, and the sum is divided by three to yield the total CES score. In this scoring system, higher values reflect a better quality of life. The validated German version of the CES was used [8].

### Statistical evaluation

For statistical analysis, PASW Statistics 25.0 (SPSS Inc., Chicago, IL, USA) was used. The distribution properties are described by arithmetic means and standard deviations.

#### Sample size justification

The enrolled sample of *n =* 79 COM patients provided adequate statistical power for planned psychometric analyses. According to COSMIN guidelines [14], exploratory factor analysis requires a minimum of 50 participants with ideal subject-to-item ratio of ≥ 5:1 to ensure stable factor extraction. Our sample achieved a ratio of 6.6:1 (79 patients/12 items), exceeding this threshold and aligning with the original COMQ-12 validation study (*n =* 120 patients) [6]. This sample size provides sufficient power to detect medium to large effect sizes in validity and responsiveness measures (α = 0.05, two-tailed; power > 0.80 for *r ≥* 0.30). Sampling adequacy was confirmed via the Kaiser–Meyer–Olkin (KMO) measure, with values ≥ 0.60 considered acceptable for factor analysis.

To evaluate the structural validity and dimensionality of the German COMQ-12, an exploratory factor analysis was performed. Sampling adequacy was assessed using the Kaiser–Meyer–Olkin (KMO) measure and Bartlett’s test of sphericity. A Principal Component Analysis (PCA) was conducted to extract the underlying components. Based on the scree plot and theoretical considerations regarding the instrument’s design, a one-factor solution was investigated to confirm the unidimensionality of the total score. Factor loadings of ≥ 0.40 were considered significant.

The reliability of the measurement instrument was evaluated through two complementary approaches. First, internal consistency was assessed via Cronbach’s α coefficient for the total score. Following established psychometric standards, instruments were considered acceptable for research use only when Cronbach’s α reached a minimum threshold of ≥ 0.65 [15].

The test–retest reliability was subsequently examined to evaluate the temporal stability of the instrument. All participants completed the COMQ-12 questionnaire on two occasions during the postoperative period: initially at the scheduled follow-up consultation and again one week later. This postoperative timing (6 months + 1 week) was chosen as clinical stability is typically achieved by this phase post-COM surgery, minimizing true change while capturing reliable repeated measures [6]. The temporal consistency between these two measurement points was analyzed via Pearson’s product‒moment correlation coefficient. Correlation values ranging from 0.70 to 0.95 were interpreted as indicating high to very high test–retest reliability [16].

Linear regression was used to analyze the relationships among the total COMQ-12, CES, and COMOT-15 scores and assess convergent validity. Discriminant validity was determined by comparing the COMQ-12 scores of COM patients with those of an ear-healthy control group via a t test. Responsiveness was described through the standardized response mean (SRM), which is defined as the quotient of the mean change and the standard deviation of the change [17]. Values ≥ 0.8 are considered large effects; values ≥ 0.5 and < 0.8 are considered medium effects; values ≥ 0.2 and < 0.5 are considered small effects; and values < 0.2 are considered minor effects [17].

A potential response shift was examined via a paired t test as an indirect method of change measurement, whereby participants provided a retrospective assessment of their initial state at the second measurement point (then test). The standardized effect size (SES) was calculated to quantify the magnitude of the observed effect, with the fundamental principle that the mean difference between two measurement points must be standardized relative to a measure of dispersion [18]. For the DES calculation, the preoperative standard deviation serves as the standardization denominator. The effect size interpretation follows Cohen’s established classification system [17], where values of approximately 0.2 represent small effects, 0.5 indicates medium effects, and 0.8 denotes large effects. Furthermore, effect sizes of ≥ 0.5 are considered clinically relevant [18, 19].

Statistical significance was set at *p < *0.05.

## Results

### Patient characteristics

The study included 79 patients with COM. At the 6-month follow-up examination. Data from 59 patients were collected, which corresponds to a response rate of 75%. Among these patients, 30 had cholesteatoma, and 29 had chronic suppurative otitis media (mucosal disease). The demographic parameters of the patients are shown in Table 1.

**Table 1:** Demographic and audiometric data of the included patient groups

		Patients (*n =* 79)
Age		48,6 ± 14,9 (SD) Years
Gender	Female	50 (63%)
	Male	29 (37%)
Pathology	Chronic suppurative otitis media	41 (52%)
	Cholesteatoma	38 (48%)
Hearing function	Air conduction affected ear (0,5–4 kHz)	48.09 ± 20.05 dB
	Air bone gap affected ear (0,5–4 kHz)	24.25 ± 14.49 dB
	Air conduction contralateral ear (0,5–4 kHz)	24.76 ± 19.83
	Air bone gap contralateral ear (0,5–4 kHz)	7.68 ± 9,50

The ear healthy control group consisted of 30 individuals with a mean age of 30.9 ± 8.1 years. The sex distribution was similar to that of the patient group, comprising 18 females (60%) and 12 males (40%; *p =* 0.82). The control group was significantly younger (mean age 30.9 ± 8.1 years vs. 48.6 ± 14.9 years in patients, *p < *0.001), which may introduce age-related bias in discriminant validity. However, this mirrors real-world otologic controls and aligns with prior COMQ-12 validations.

The enrolled sample of *n =* 79 patients provided a subject-to-item ratio of 6.6:1 (79/12 items), exceeding the COSMIN minimum requirement of ≥ 5:1 for exploratory factor analysis. This sample size is comparable to the original COMQ-12 validation (*n =* 120 patients) and ensured adequate statistical power for all planned psychometric analyses. Specifically, power analysis confirmed > 0.80 power to detect medium to large effect sizes (*r ≥* 0.30) for validity correlations at α = 0.05 (two-tailed). The Kaiser–Meyer–Olkin (KMO) measure of 0.82 further confirmed that sample size was sufficient for factor analysis (minimum threshold ≥ 0.60). Additionally, the achieved internal consistency estimates (Cronbach’s α ≥ 0.84) and test–retest reliability (r = 0.89) demonstrated stable and reliable measurement without inflation of error variance, supporting the adequacy of *n =* 79 for this validation study.

### Factor analysis and structural validity

The exploratory factor analysis confirmed the suitability of the data for structure detection (KMO = 0.82; Bartlett’s test of sphericity: χ^2^(66) = 426.76, *p < *0.001). A Principal Component Analysis (PCA) extracted a single component with an eigenvalue of 5.13, explaining 42.74% of the total variance. The scree plot supported this one-factor solution, showing a clear “elbow” after the first component.

All 12 items of the COMQ-12 demonstrated high factor loading on this single component, ranging from 0.46 to 0.77 (Table 2). These results support the unidimensionality of the German COMQ-12 and justify the calculation of a single total score for clinical and research purposes.

**Table 2:** Factor loadings from Principal Component Analysis (PCA) of the German COMQ-12 (*n =* 79)

Item	Content (short)	Factor Loading
1	Ear discharge	0.66
2	Smelling ear discharge	0.66
3	Hearing at home (TV/Radio)	0.77
4	Hearing in groups	0.70
5	Hearing on phone	0.55
6	Vertigo/Dizziness	0.46
7	Tinnitus	0.56
8	Ear discomfort/pain	0.73
9	Care of the ear	0.64
10 General impact	General impact on work/daily life	0.59
11	GP visits	0.72
12	Medication use	0.73

Extraction method: Principal Component Analysis; 1 component extracted explaining 42.74% of variance

### Psychometric characteristics of the German COMQ-12 version

The COMQ-12 showed high internal consistency both preoperatively (Cronbach’s α = 0.87) and postoperatively (Cronbach’s α = 0.84) (Table 3).

**Table 3:** Psychometric characteristics of the German-language COMQ-12 in comparison to the respective original publications

Parameter	Own data	Original data
Cronbach-α preoperative	0.87	0.89
Cronbach-α postoperative	0.84	-
Test–retest reliability (r)	0.89***	-
Responsiveness (SRM)	0.89 ***	-
Correlation (r) with the air conduction threshold (0,5–4 kHz)	0.16	0.02
Response shift (SES)	−0.30	-

^**^ *p < *0.01 very significant *** *p ≤* 0.001 highly significant; "- “ no data available, *SES* Standardized effect size, *SRM* Standardized response mean, The psychometric data of the original studies for the COMQ-12 are taken from the publication by Phillips et al. 2014 [6]

When determining test–retest reliability, a correlation coefficient of *r* = 0.89 was obtained for the total score, indicating high test–retest reliability (Table 3).

**Fig. 2:**
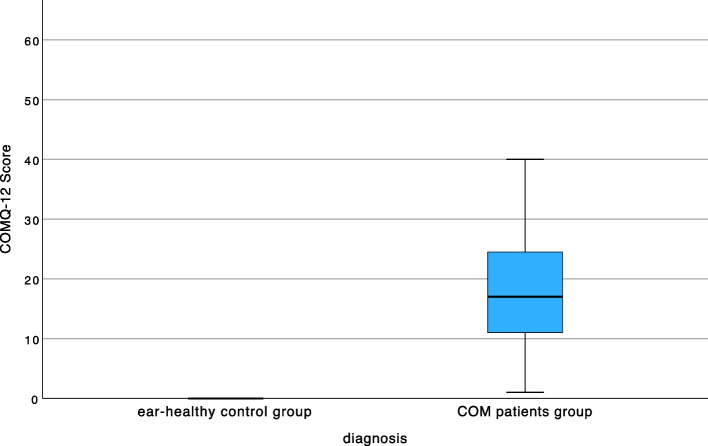
Testing the discriminant validity of the COMQ-12 between patients' preoperative data (*n =* 79) and an ear-healthy control group (*n =* 30). ***p ≤ 0.001 highly significant

To investigate discrimination ability, the German translation of the CES was also completed by an "ear-healthy" control group (*n =* 30). For all scales, the control group reported a significantly lower impairment of HRQoL than did the patient group COM (Fig. 2).

The item difficulty of the COMQ-12 (Table 4) revealed that all the items were within the recommended cutoff range. The mean item difficulty of the COMQ-12 is 32.59% (range: 17.8%—56.0%), indicating that most items are relatively challenging for responders. The item discrimination indices demonstrate satisfactory psychometric properties for the COMQ-12, with mean values of 0.57 for the COMQ-12. All item values are well above the range classified as “fair” (0.10–0.30). The proportion of highly discriminative items (≥ 0.50) favors the COMQ-12, with 75% of the items meeting this criterion.

**Table 4:** Variance, discriminatory power, and item difficulty for the items of the COMQ-12

Item	MW	SD	Variance	Discriminatory power	Item difficulty	Range
COMQ-12 (Fig. 1)						
1	1.06	1.24	1.55	0.56	26.5	0—4
2	1.08	1.47	2.17	0.55	21.6	0—5
3	2.42	1.30	1.68	0.69	48.4	0—5
4	2.80	1.26	1.57	0.60	56	0—5
5	1.29	1.31	1.72	0.49	25.8	0—5
6	0.89	1.40	1.95	0.40	17.8	0—5
7	1.75	1.68	1.83	0.48	35	0—5
8	1.00	1.54	2.39	0.68	20	0—5
9	1.39	1.74	3.04	0.55	27.8	0—5
10	2.35	1.25	1.57	0.52	47	0—5
11	1.32	1.55	2.40	0.64	26.4	0—5
12	1.94	1.62	2.62	0.63	38.8	0—5

*MW* Mean value, *SD* Standard deviation

**Fig. 3:**
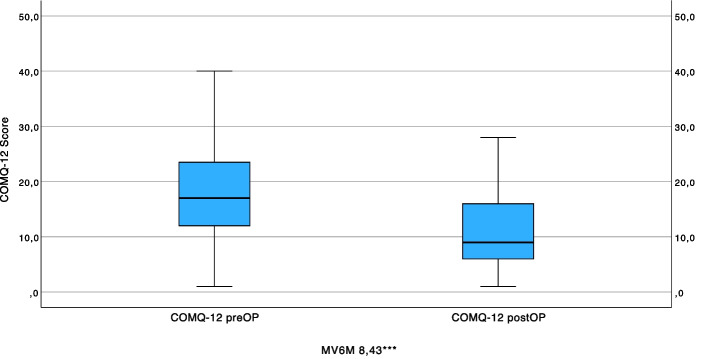
Responsiveness of the COMQ-12: pre- and postoperative comparison (*n =* 59). MV6M Mean difference in scores of CES preoperatively—6 months postoperatively, ***p ≤ 0.001 highly significant

In the pre- and postoperative comparisons (responsiveness), large improvements in HRQoL were demonstrated for the COMQ-12 (SRM = –0.80; Fig. 3). There was only a poor correlation between the postoperative total score and the postoperative air conduction threshold (*r* = 0.16). Bilateral audiological assessment revealed that the contralateral ear had substantially better hearing thresholds (mean air conduction: 24.76 ± 19.83 dB) compared to the affected ear (48.09 ± 20.05 dB), with corresponding differences in air-bone gaps (Table 1). According to WHO classification, functional hearing disability is primarily determined by the better-hearing ear, which in our cohort was the contralateral ear. This provides important clinical context for interpreting the weak correlations between COMQ-12 scores and audiometric parameters.

To test for a possible response shift, a paired t test was performed between the respective total scores of the preoperative situation. The total scores of the COMQ-12 revealed a shift toward a better quality of life (Table 3). A moderate effect size (SES = 0.30) was determined.

### Convergent validity: correlation of the German COMQ-12 with the COMOT-15 and the CES

**Fig. 4:**
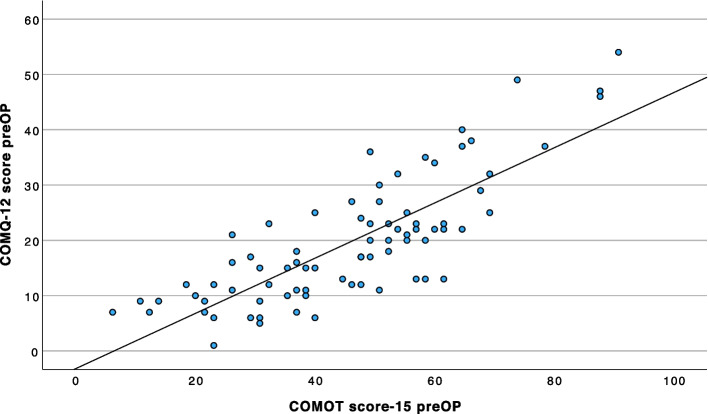
Linear regression between the preoperative total score of the COMQ-12 and the total score of the COMOT-15 (*n =* 79). A strong positive correlation (*r* = 0.81) is observed, as higher scores on both measures indicate poorer HRQoL

**Fig. 5:**
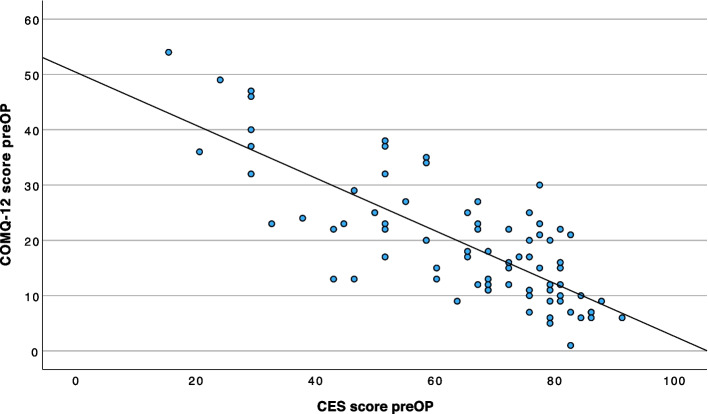
Linear regression between the preoperative total score of the COMQ-12 and the total score of the CES (*n =* 79). An inverse correlation (*r* =—0.78) is observed, as higher CES scores indicate better HRQoL, whereas higher COMQ-12 scores reflect poorer HRQoL

There is a strong correlation between total COMQ-12 scores and COMOT-15 scores (*r* = 0.81, 95% CI 0.71—0.87; Fig. 4), as well as between total COMQ-12 scores and CES scores (*r* =—0.78, 95% CI −0.85—0.67; Fig. 5).

The following equations were obtained via linear regression to model the relationship between the COMQ-12 and COMOT-15 preoperative total scores:

- Total score COMQ-12 = 0.49 × total score COMOT-15–3.18.
- Total score COMOT-15 = 1.30 × total score COMQ-12 + 20.17.

The following equations model the relationship between the COMQ-12 score and preoperative CES score:

- Total score COMQ-12 = −0.47 × total score CES + 50.38.
- Total score CES = −1.27 × total score COMQ-12 + 89.55.

## Discussion

### Development and structural characteristics of the COMQ-12

The Chronic Otitis Media Questionnaire 12 (COMQ-12) was introduced in 2014 as a concise, disease-specific instrument for assessing health-related quality of life (HRQoL) in individuals with chronic otitis media (COM) [6]. Its development was based on a methodical reduction and synthesis of items from three previously validated instruments: the Chronic Ear Survey (CES), the Chronic Otitis Media Outcome Test 15 (COMOT-15), and the Chronic Otitis Media-5 (COM-5). Of the twelve items comprising the COMQ-12, seven directly address otologic symptoms such as otorrhea, hearing loss, tinnitus, and vertigo. This symptom-oriented focus underscores the instrument’s utility in capturing physical disease burden but offers comparatively limited insight into the social and psychological dimensions of HRQoL [6].

The COMQ-12 differs from multidimensional instruments such as the COMOT-15 or the CES in that it yields a single total score without subscores. While this limits domain-specific interpretability, it simplifies administration, enhances feasibility for routine use, and reduces the risk of statistical error due to multiple testing. These properties make the instrument particularly suitable for large-scale or cross-cultural applications.

While some validation studies have explored multi-factorial solutions for the COMQ-12, such as a three-factor structure distinguishing between ear symptoms, hearing and activity limitations [5], our data strongly supported a unidimensional structure, which fulfills the COSMIN standard for structural validity. The exploratory factor analysis revealed a dominant single component explaining 42.74% of the variance, with all 12 items loading significantly on this component, providing robust evidence for the instrument’s internal structure. From a clinical perspective, this unidimensionality validates the use of the total score as a single metric for overall disease burden in accordance with COSMIN requirements for construct validity, facilitating straightforward interpretation in daily practice without complex subscale calculations. While PCA confirmed unidimensionality, confirmatory factor analysis (CFA) was not performed due to sample size constraints (*n =* 79); future larger-scale validations should include CFA.

### Psychometric properties: reliability and validity

The original validation of the COMQ-12 involved 50 adult patients with COM and focused exclusively on pretreatment assessment [6]. The reported internal consistency (Cronbach’s α = 0.88) was high and closely mirrored the present German validation, which yielded values of 0.87 preoperatively and 0.84 postoperatively. The current study also extended the psychometric evaluation by including test‒retest reliability—a property not assessed in the original publication. The German version demonstrated excellent temporal stability, with a Pearson correlation coefficient of *r* = 0.89 (*p < *0.001), thus confirming its reliability in repeated measures.

The construct validity of the original COMQ-12 was established via expert review and comparisons between clinically distinct patient groups [6]. Similarly, the German version demonstrated strong discriminant validity, as evidenced by significantly lower scores in an otologically healthy control group than in the COM cohort.

### Responsiveness and suitability for longitudinal assessment

A notable contribution of the present study lies in the assessment of responsiveness, a dimension not addressed in the original COMQ-12 validation. The German version showed a standardized response mean (SRM) of –0.80, indicating a large effect size and confirming the instrument’s sensitivity to clinically meaningful changes after surgical intervention [20]. These findings establish the COMQ-12 as a suitable tool not only for cross-sectional assessment but also for longitudinal monitoring of treatment outcomes.

In this context, it is particularly relevant to note that the COMQ-12, despite being originally validated for single-time use, has characteristics that make it preferable to retrospective assessment instruments. Tools such as the Glasgow Benefit Inventory (GBI) [21] or the Chronic Otitis Media Benefit Inventory (COMBI) [22] rely on post hoc evaluations of the preoperative condition and are thus prone to recall bias and subjective reinterpretation of past health states. In contrast, the COMQ-12 permits direct, prospective measurement of HRQoL at multiple time points, which improves internal validity and provides a more accurate depiction of treatment effects over time.

The impact of a response shift—a psychological phenomenon in which patients reevaluate their internal standards following therapeutic interventions—was also investigated. The effect observed for the COMQ-12 (SES = 0.30) was small and not clinically significant, further supporting the tool’s robustness for longitudinal application.

### Strengths and limitations of this study

This validation study benefits from several methodological strengths. The prospective design with standardized data collection at predefined time points (preoperatively, 6 months postoperatively) minimizes recall bias and strengthens internal validity. The study adhered to COSMIN guidelines for PROM validation, implementing comprehensive psychometric evaluation including internal consistency, test–retest reliability, discriminant validity, convergent validity, responsiveness, and response shift assessment. The translation process employed forward–backward translation combined with expert review and cognitive debriefing with patients, ensuring linguistic and cultural equivalence of the German version.

However, several limitations merit consideration. While the sample size of *n =* 79 meets COSMIN requirements for structural validity and exploratory factor analysis, it may limit generalizability to specific patient subgroups such as revision surgery cases or patients with severe ossicular erosion. The study was conducted at a single tertiary referral center, which may restrict generalizability to other healthcare settings with different surgical practices or patient populations. The 6-month follow-up response rate was 75% (59/79); although acceptable, potential attrition bias cannot be entirely excluded. A key limitation is the age disparity between patients (48.6 years) and controls (30.9 years), potentially inflating discriminant validity due to younger adults reporting fewer symptoms. Future studies should age-match groups. Responsiveness was assessed only at 6 months; lack of long-term follow-up (e.g., 12—24 months) limits insights into sustained effects. Detailed surgical technique analysis was not performed as multiple COM surgery options would have resulted in subgroups to small for meaningful analysis (n < 20 expected per technique).

### Correlations with audiometric parameters and comparative interpretation

Consistent with findings from the original study [6], the present data revealed only weak correlations between COMQ-12 scores and audiometric parameters. This was expected given that auditory function is addressed in only two of the twelve COMQ-12 items. Similar patterns have been observed for other disease-specific PROMs, including the COMOT-15 and CES [7–9], and emphasize the limited capacity of PROMs to reflect purely objective hearing thresholds [23]. Consequently, audiometric and patient-reported data should be viewed as complementary rather than interchangeable. Moreover, functional hearing disability is primarily determined by the better-hearing ear, which in our cohort was typically the contralateral side; this clinical context may further explain why correlations between COMQ-12 scores and affected-ear audiometric thresholds were only weak.

As expected from item overlap, strong correlations (*r* = 0.81 vs. COMOT-15; *r* = 0.78 vs. CES) validate conceptual alignment and enable instrument conversion. Such methods could facilitate meta-analyses or multicenter collaborations, particularly in the absence of a unified standard for HRQoL measurement in otologic surgery.

Although the COMQ-12 also incorporated elements from the COM-5 during its development, a direct psychometric comparison was not feasible in this study. The COM-5 is a pediatric-specific instrument and has not been validated in adult populations, rendering any direct comparison inappropriate in this context [10].

### Clinical and research implications

To date, there is no internationally standardized PROM for use in adult patients undergoing surgery for chronic otitis media. This has led to heterogeneous use of instruments across countries and studies, complicating the comparability of outcomes. Within this landscape, the COMQ-12 represents a compelling candidate for broader adoption. It has been translated and validated in numerous languages, including Dutch, Portuguese, Serbian, and Russian, and has gained considerable international traction.

With strong psychometrics and international comparability, the German COMQ-12 outperforms linguistically limited alternatives like COMOT-15 and outdated CES for clinical/research use.

## Conclusion

The German COMQ-12 is a reliable, valid, and responsive tool for measuring symptom-related quality of life in patients with chronic otitis media. Its simplicity, international comparability, and suitability for longitudinal assessment render it a valuable tool in both routine care and outcome research.

## Data Availability

The data that support the findings of this study are not publicly available as they form part of a larger, ongoing research project that has not yet been completed. The dataset used in this study constitutes a subset of a comprehensive data collection. Data may be available from the corresponding author upon reasonable request and with permission from the research team overseeing the lager study.
